# Reply to Datta, “Is static or cidal antibiotic falsifiable?”

**DOI:** 10.1128/aac.01391-25

**Published:** 2025-10-13

**Authors:** Brad Spellberg, Noah Wald-Dickler, Paul Holtom, Pascal Meyer-Sautter, Austin Camp, Alejandro Diaz Diaz, Ranya Buhamad, Ali Sebastian Meza Vazquez, Gloria Mayela Aguirre-Garcia, Matthew Stanton, Susan M. Butler-Wu, Isabelle Chiu, Zeynep Ergenc, Guru Bhoojhawon, Rita Murri, Alberto Enrico Maraolo, Gabriela Cabanilla, Niccolò Riccardi, Vhudzani Tshisevhe, Curtis Behenna, Karen S. Williams, Wesley D. Kufel, Natalia Wojciaczyk, Bernardo Vidal Pimentel, Ahmed Muyidi, Rodrigo P. L. Costa, Fabrizio Motta, Émilie Bortolussi-Courval, Todd C. Lee, Emily G. McDonald, Bassam Ghanem, Zachary Nelson

**Affiliations:** 1 Los Angeles General Medical Center 23336 , Los Angeles, California, USA; 2 Department of Internal Medicine, Kantonsspital Glarus, Glarus, Switzerland; 3 Union University College of Pharmacy, Jackson, Tennessee, USA; 4 Department of Pediatric Infectious Diseases, Hospital Pablo Tobón Uribe, Hospital General de Medellín 576742 , Medellín, Colombia; 5 Jaber Al Ahmad Al-Sabah Hospital, Ministry of Health 698084 , Kuwait, Kuwait; 6 Department of Internal Medicine, Mexican Institute of Social Security, Western National Medical Center, High Specialty Medical Unit, Guadalajara, Mexico; 7 Tecnologico de Monterrey, Escuela de Medicina y Ciencias de la Salud 70656, Monterrey, Mexico; 8 Medical College of Wisconsin School of Pharmacy, Froedtert and the Medical College of Wisconsin, Milwaukee, Wisconsin, USA; 9 Division of Infectious Diseases, University of Alberta, Edmonton, Alberta, Canada; 10 Vaccine Institute, City St George's, University of London, London, United Kingdom; 11 Paediatric Infectious Diseases and Immunology, Great Ormond Street Hospital for Children, London, United Kingdom; 12 Division of Pediatric Hospital Medicine, Driscoll Children’s Hospital, Corpus Christi, Texas, USA; 13 Infectious Diseases Unit, Fondazione Policlinico Gemelli IRCCS, Rome, Italy; 14 Section of Infectious Diseases, Department of Clinical Medicine and Surgery, University of Naples "Federico II", Naples, Italy.; 15 Department of Pharmacy, Division of Infectious Diseases, Department of Internal Medicine, University of New Mexico Health Sciences Center 12289, Albuquerque, New Mexico, USA; 16 TB Reference Centre and Laboratory, ASST Grande Ospedale Metropolitano Niguarda 9338, Milan, Italy; 17 Lancet Laboratories 518386 , Rustenburg, South Africa; 18 Department of Medical Microbiology, University of Pretoria 574011, Pretoria, South Africa; 19 Department of Infectious Disease, Ascension St John Medical Center 159297 , Tulsa, Oklahoma, USA; 20 Department of Pharmacy, Guthrie Robert Packer Hospital, Sayre, Pennsylvania, USA; 21 School of Pharmacy and Pharmaceutical Sciences, State University of New York at Binghamton, Binghamton, New York, USA; 22 State University of New York Upstate Medical University, Syracuse, New York, USA; 23 The Guthrie Clinic, Sayre, Pennsylvania, USA; 24 Department of Internal Medicine, Hospital da Luz Lisboa 199396 , Lisbon, Portugal; 25 King Faisal Medical City, Riyadh, Saudi Arabia; 26 Hospital Mater Dei Salvador 223018, Salvador, Brazil; 27 Hospital Cardio Pulmonar da Bahia 534569 , Salvador, Brazil; 28 Division of Infectious Diseases, Santa Casa de Porto Alegre, Porto Alegre, Brazil; 29 Faculté de Médecine et des sciences de la santé, Université de Sherbrooke 7321, Sherbrooke, Canada; 30 Division of Infectious Diseases, Department of Medicine, McGill University, Montreal, Canada; 31 Division of General Internal Medicine, Research Institute of the McGill University Health Centre 507266, Montreal, Canada; 32 Pharmaceutical Care Department, King Abdulaziz Medical City, National Guard Health Affairs, Jeddah, Saudi Arabia; 33 HealthPartners and Park Nicollet Health Services, Minneapolis, Minnesota, USA; Columbia University Irving Medical Center, New York, New York, USA

## REPLY

We would like to thank Dr. Datta (1) for his interest in our article (2). Dr. Datta wishes to defend the static vs. cidal antibiotic model against “absolutist framing.” He invokes “mono-parametric” assays like “MBC/MIC ratios or…reactive oxygen species generation” to suggest that *in vitro* static vs. cidal phenomenology may be phenotypically real.

However, MBC/MIC ratios and reactive oxygen species generation have never been demonstrated to predict different efficacy *in vivo*. Indeed, as we summarized, 60 randomized controlled trials (RCTs) definitively demonstrated that the static/cidal framing of antibiotics specifically does not predict differences in clinical efficacy (2). RCTs are the most reliable means of dealing with complexity when evaluating the effect of an intervention (i.e., a drug). Ironically, the points Dr. Datta raised actually reinforce our central message: *in vivo* clinical efficacy depends on pharmacokinetic/pharmacodynamic parameters, site of infection, immune status, and tissue penetration, and not on the static/cidal classification of an antibiotic.

More fundamentally, as we emphasized in our letter, static antibiotics do kill bacteria, contrary to the connotation of the term “static.” The term “bacteriostatic” originated in the 1920s, before even sulfonamides were available, based on crude, macroscopic visualization of the effect of colored dyes on bacterial colonies (3). The term was never subsequently updated, such that it is still incorrectly used to imply that an antibiotic inhibits bacterial replication without killing the bacteria.

Thus, irrespective of any assays based on “mono-”, “multi-”, or any other Latin multiple of “-parametric,” we reiterate that the terms static and cidal are archaic, misleading, and incorrect. Furthermore, given their tendency to push clinicians toward potentially harmful care decisions—such as adding aminoglycosides to improve a presumed “cidal effect,” or refusing to use static linezolid to treat bacteremia or endocarditis despite compelling data demonstrating its clinical efficacy (4–6)—this classification should be abandoned.

Finally, Datta quotes the philosopher Popper to make the point that the hallmark of a scientific hypothesis is falsifiability and suggests that we have not left the question of static/cidal open to future evidence. However, given how often *in vitro* constructs have failed to translate to clinical efficacy, the burden of proof must shift to those who wish to advance the relevance of *in vitro* paradigms. Indeed, Popper also said, “Every scientific theory must be subjected to severe tests; it must be put to the test to see whether it withstands attempts at falsification.” (7). That is precisely what we did, thereby demonstrably falsifying the static/cidal theorem via rigorous investigation.

Thus, the static vs. cidal myth should be retired until new, compelling data become available that somehow are able to supersede 60 concordant RCTs demonstrating that it is of no clinical relevance. And even in the highly unlikely event that were to occur, the term “static” would need to be rebranded so as to avoid the false implication that the antibiotic inhibits cell division without killing the bacteria.
